# Screening, purification and characterization of thermostable, protease resistant Bacteriocin active against methicillin resistant *Staphylococcus aureus* (MRSA)

**DOI:** 10.1186/s12866-018-1337-y

**Published:** 2018-11-22

**Authors:** Asma Ansari, Rashida Rahmat Zohra, Omer Mukhtar Tarar, Shah Ali Ul Qader, Afsheen Aman

**Affiliations:** 1 The Karachi Institute of Biotechnology & Genetic Engineering (KIBGE)University of Karachi, 75270, Karachi, Pakistan; 2 Jinnah University for Women, Karachi, Pakistan; 3 Food and Marine Resources Research Centre, Pakistan Council of Scientific & Industrial Research (PCSIR), Laboratories Complex Karachi, 75280, Karachi, Pakistan; 4 Department of Biochemistry, University of Karachi, 75270, Karachi, Pakistan

**Keywords:** Antibacterial, Potential/*Bacillus*, *subtilis*/Bacteriocin, Purification/ multidrug, Resistance/Thermostable

## Abstract

**Background:**

The emergence of serious issues of multidrug resistance in the past few years have enforced the use of bacteriocins for combating infections. Threat posed to public health by various multidrug resistant (MDR) organisms can be resolved by discovering new antimicrobial proteins with broad spectrum of inhibition.

**Results:**

In the current study, Bacteriocin (BAC-IB17) produced by *Bacillus subtilis* KIBGE-IB17 is found to be effective against different strains of methicillin resistant *Staphylococcus aureus* (MRSA). The approximate molecular mass of BAC-IB17 is 10.7 kDa. This unique bacteriocin is found to be highly thermostable and pH stable in nature. It also showed its stability against various heavy metals, organic solvents, surfactants and proteolytic enzymes. Amino acid profile of BAC-IB17 clearly showed that this protein mainly consists of non-polar and basic amino acids whereas; some acidic amino acids were also detected. Sequence of first 15 amino acid residues obtained from N-terminal sequencing of BAC-IB17 were NKPEALVDYTGVXNS.

**Conclusions:**

The anti-MRSA property of purified bacteriocin may be used to prevent the spread of MRSA infections. Remarkable features of BAC-IB17 suggests its applications in various pharmaceutical and food industries as it can function under a variety of harsh environmental conditions.

## Background

Numerous concerns have been raised against increased bacterial resistance towards effective drugs and become a debated issue all over the world. Now a days, there is an increase consumer demands for the natural antimicrobial compounds to overcome emergence of multidrug resistance. Microorganisms are involved in the production of an astonishing range of natural antimicrobial compounds as their defense mechanism. In the past few years, research on bacteriocins is opening a door of a new age as a new generation of antimicrobials. Therefore, threat posed to public health by multidrug resistant organisms prevalent in the community can be resolved by the discovery of new antimicrobial proteins with broad spectrum of inhibition.

Methicillin resistant *Staphylococcus aureus* (MRSA) is an alarming threat of interest that is responsible for either community acquired (CA-MRSA) or health-care-acquired (HA-MRSA) infections. MRSA is involved in several lives threatening human infections as it is resistant to almost all β-lactam antibiotics and now a days responsible for the emerging cases of MDR [1–5]. The aforesaid problem is persisting not only because of the development of different ways to resist effective antibiotics by multidrug resistant microorganisms however, incessant misuse of antibiotics in human medicines, veterinary and agricultural sectors are also predominantly contributing in this phenomenon. Therefore, for the effective control of MDR organisms, it is imperative to discover and characterize new antibacterial proteins. Bacteriocins produced by Gram’s positive bacteria exhibited a broad spectrum of inhibition as compared to other bacteriocins.Most of the species from genus *Bacillus* are generally recognized as safe (GRAS) to produce antimicrobial compounds for the treatment of infections in humans [6–10]. *Bacillus subtilis* probiotics and their metabolites are promising candidates in biotechnological applications including production of enzymes, amino acids, antifungal and antibacterial substances. All these factors increase the industrial importance of this organism with the reduction in potential risk factors associated with the use of *B. subtilis* in industries. The plasmid borne bacteriocin (BAC-IB17) expressed and secreted by a locally isolated strain of *Bacillus subtilis* KIBGE-IB17 [GenBank: HQ588347] showed broad spectrum of inhibition against various pathogenic and non-pathogenic strains [11, 12]. The current study hypothesize that BAC-IB17 are able to suppress the MRSA growth and may be successful as an alternative therapeutic agent to prevent spread of MDR organisms. The aim of the current study was to purify, characterize and to elucidate the properties and the antibacterial potential BAC-IB17 against MDR organisms. Therefore, a pool of effective drugs could be available to overcome the newly emerging drug resistant bacteria.

## Methods

### Bacterial strains

The sensitive strains used in the current study were isolated from clinical, soil and water samples collected from different localities in Karachi, Pakistan and no specific permission was required for the isolation of microbial strains because the isolated strains are indigenous in the local environment and are not endangered or protected species (Table 1). Isolates obtained from primary culture were grown on non-selective agar and identified based on morphological, biochemical characteristics and 16S rDNA gene sequencing [13]. For the specific confirmation of MRSA and MSSA, sensitivity test was performed using methicillin and bacitracin. Whereas, Gram’s reaction, pigment production, catalase test, coagulase test, oxidase test, citrate utilization, nitrate reduction, growth in NaCl and sugar fermentation (mannitol and glucose) were also conducted. 16S rDNA gene sequences of MRSA and MSSA were analyzed by similarity search using BLAST (http://www.ncbi.nlm.nih.gov/BLAST/). Percentage identity between the current isolates and the previously reported 16S rDNA sequences which were available in the GenBank database was constructed using Clustal-W and MegAlign programmes (Lasergene, DNASTAR Inc., Madison, USA).

**Table 1:** Inhibitory spectrum of BAC-IB17 against different bacterial strains

Indicator strains	Source	Temperature (°C)	Incubation time (hrs)	Sensitivity^**a**^
*Staphylococcus aureus* (MRSA)	Clinical (KIBGE-IB23)	37	24	++++
*Staphylococcus aureus* (MSSA)	Clinical (KIBGE-IB24)	37	24	++++
*Staphylococcus aureus* (MSSA)	Water	37	24	++++
*Staphylococcus aureus* (MSSA)	Soil	37	24	++++
*Staphylococcus aureus* (MRSA)	Clinical Isolate	37	24	+++
*Staphylococcus aureus* (MRSA)	Clinical Isolate	37	24	+++
*Staphylococcus aureus* (MRSA)	Clinical Isolate	37	24	+++
*Enterococcus faecalis*	Soil	37	48	+++
*Staphylococcus aureus* (MRSA)	Clinical Isolate	37	24	++
*Staphylococcus aureus* (MRSA)	Clinical Isolate	37	24	++
*Bacillus stearothermophilus*	Soil (KIBGE-IB29)	60	24	++
*Bacillus subtilis*	Soil	37	24	++
*Listeria monocytogenes*	ATCC 7644	35	48	++
*Staphylococcus epidermidis*	Environment	37	24	++
*Salmonella typhi A*	Clinical Isolate	37	24	+
*Staphylococcus aureus* (MRSA)	Clinical Isolate	37	24	+
*Salmonella typhimurium*	Clinical Isolate	37	24	+
*Escherichia coli*	ATCC 8739	35	18	+
*Bacillus licheniformis*	Environment (KIBGE-IB2)	40	24	+
*Bacillus licheniformis*	Soil (KIBGE-IB4)	40	24	–

^**a**^**Key**: (−) No Antgonistic Activity; (+) Inhibitory zone diameter within 11-15 mm; (++) Inhibitory zone diameter within 16-20 mm; (+++) Inhibitory zone diameter within 21-25 mm; (++++) Inhibitory zone diameter within 26-30 mm

### BAC-IB17 production and purification

Production of BAC-IB17 was augmented by manipulating a variety of physical and chemical parameters [11]. Extracellular bacteriocin was obtained after centrifugation of 24 h grown culture medium at 35,000 ×*g* for 15 min at 4 °C. Cell free supernatant containing BAC-IB17 was filtered through 0.22 μm filter membrane (Millipore, USA) using a filtration assembly (Sartorius, Germany) under sterile conditions for further studies. Bacteriocin was partially purified by gradient salt precipitation method. Partially purified bacteriocin was desalted using PD-10 desalting column with a cutoff value of 5.0 kDa and filtered using Centricon Ultracel YM-10 and YM-30 centrifugal filter device. Gel permeation chromatography was performed for the purification of the BAC-IB17. All the collected fractions were assayed for antibacterial activity and the fractions that showed anti MRSA activity were pooled and freeze dried [14]. This purified BAC-IB17 was used for further characterization purpose.

### Calculation of arbitrary units and minimal inhibitory concentration (MIC)

The antimicrobial activity of bacteriocin was detected by agar well diffusion method [15]. Two-fold serial dilutions of purified bacteriocin was prepared and the activity was expressed in terms of arbitrary units per milliliter (AU ml^− 1^). One arbitrary unit (AU) is defined as the reciprocal of the highest dilution of bacteriocin showing a clear zone of growth inhibition of indicator strain.

For the calculation of arbitrary units of bacteriocin, twofold serial dilutions were prepared in 50.0 mM potassium phosphate buffer (pH -7.0). Each dilution (100.0 μL) was placed into the wells on nutrient agar plate which was previously spreaded with the indicator strain. The plate was incubated at 37 °C for 24 h and the zone of inhibition was measured in millimeters. Arbitrary units were calculated using the following formula:

$$ \boldsymbol{Arbitrary}\ \boldsymbol{Units}\ \left(\boldsymbol{AU}/\boldsymbol{ml}\right)=\frac{\boldsymbol{Reciprocal}\ \boldsymbol{of}\ \boldsymbol{the}\ \boldsymbol{Highest}\ \boldsymbol{Dilution}\ }{\boldsymbol{Amount}\ \boldsymbol{of}\ \boldsymbol{Bacteriocin}\ \boldsymbol{Used}}\times \mathbf{1000} $$

MIC is measured using tube dilution method. Twofold serial dilutions of purified bacteriocin with initial concentration of 0.43 mg ml^− 1^ were prepared in 50.0 mM potassium phosphate buffer (pH -7.0). A fixed volume (100.0 μL) of standardized inoculum (10^8^ CFUml^− 1^) of the indicator organism was incorporated in each dilution tube. The final concentration of bacteriocin preparation is become one-half of the initial concentration in each tube. Tubes were incubated at 37 °C for 24 h. The tubes were examined for any visible sign of bacterial growth by plate counting method. The highest dilution without bacterial growth is considered as the minimal inhibitory concentration of BAC-IB17. Total protein concentration of the samples was calculated by Lowry’s method using bovine serum albumin as standard [16].

### Killing kinetics of BAC-IB17

To determine the killing kinetics of BAC-IB17, indicator strains (logarithmic phase) were harvested by centrifugation and suspended into fresh nutrient broth. BAC-IB17 preparation (80 AU ml^− 1^) with the protein concentration of 0.05 mg ml^− 1^ was added to the indicator organisms (10^8^ CFUml^− 1^) and incubated at 37 °C for 24 h. Samples were drawn at different time intervals and plated on nutrient agar plates to determine the survival rate of each indicator organism. Optical density at 600 nm of the samples was also measured. Indicator organisms alone were used as a control.

### BAC-IB17 characterization

#### Molecular weight estimation

Tricine SDS-PAGE was performed to determine the approximate molecular weight of the bacteriocin by comparing its electrophoretic mobility with the mobility of standard proteins of known low molecular weight markers (M.W. 6500–66,000 Da; M3913, Sigma-Aldrich). Electrophoresis was carried out according to the method as described by Schägger [17] with some modifications and for the confirmation of the band of interest, gel overlay assay was performed.

#### Thermal and pH stability of BAC-IB17

Different physical and chemical parameters were considered in order to determine the kinetic behavior of BAC-IB17 (0.05 mg ml^− 1^) with respect to its clinical applications. Thermal stability of bacteriocin was performed at various temperatures ranging from 40 °C to 100 °C for different time intervals. For pH stability, bacteriocin was treated with various buffers having different pH values ranging from 3.0 to 9.0 for 2.0 h at 37 °C. Sodium citrate buffer (50 mM, pH: 3.0 to 6.0); potassium phosphate buffer (50 mM, pH: 7.0) and TrisHCl buffer (50 mM, pH: 8.0 to 9.0) were used. Buffers alone were used as negative controls. After treatments, agar well diffusion assay was performed and plates were incubated at 37 °C for 24 h and percent relative activity was calculated. For the determination of storage stability, BAC-IB17 was stored at − 20 °C for 01 year.

#### Effect of metal ions, surfactants and organic solvents on BAC-IB17

All metal ions were used as chloride salts (1.0 mM, 5.0 mM and 10 mM). BAC-IB17 was pre-incubated with metal ions, surfactants and organic solvents before antibacterial activity assay at 37 °C for 1 h and the percent relative activity was compared with reference to control.

#### Effect of enzymes on antibacterial potential of BAC-IB17

BAC-IB17 was also treated with various enzymes at 37 °C and 50 °C up to 24 h including protease (MP Biomedicals, USA), proteinase-K (Life Technologies, USA), amylase (Sigma-Aldrich, USA), pepsin, lysozyme, ribonuclease A and catalase (Serva, Germany). For this 1.0 mL of purified bacteriocin with initial concentration of 0.43 mg ml^− 1^ was incubated with equal volume of enzymes with the final concentration of all the enzymes used was 1.0 mg ml^− 1^ and the relative antibacterial activity was calculated using agar well diffusion assay.

#### Comparison of BAC-IB17with different antibacterial drugs

The effect of bacteriocin on MRSA was evaluated with reference to different drugs available in the market. For this purpose, different antibiotics including vancomycin, teicoplanin, linezolid, cefepime, ceftriaxone, ertapenem, ampicillin, ciprofloxacin, clindamycin and oxacillin were purchased from the local vendor (Oxoid Ltd., Hampshire, UK) and their minimal inhibitory concentrations (MICs) were compared with the MIC of the BAC-IB17 using agar well diffusion assay. The minimal inhibitory concentration of the BAC-IB17 used in this experiment was 0.05 mg ml^− 1^.

#### Relative amino acid composition analysis and N-terminal sequencing of BAC-IB17

Amino acids analysis was performed using O-phthalaldehyde (OPA) derivatization through an amino acid analyzer (Shimadzu LC -10A/C - R7A, USA). Purified bacteriocin was blotted on a polyvinylidenedifloride (PVDF) membrane using a semi-dry blotting device (Thermo Scientific, USA). Electroblotting was performed by the method as described earlier with some modifications [18]. The sample was sent to the protein-sequencing facility at Alta Biosciences Limited (Birmingham, United Kingdom) for the determination of N-terminal sequence. The sample was analyzed directly by using an ABI Procise 491 Protein Sequencer.

All the experiments were performed in triplicate and the results are the mean of all observations with the standard error ± 3.0 AU.

## Results

### Screening and inhibitory spectrum

**Fig. 1:**
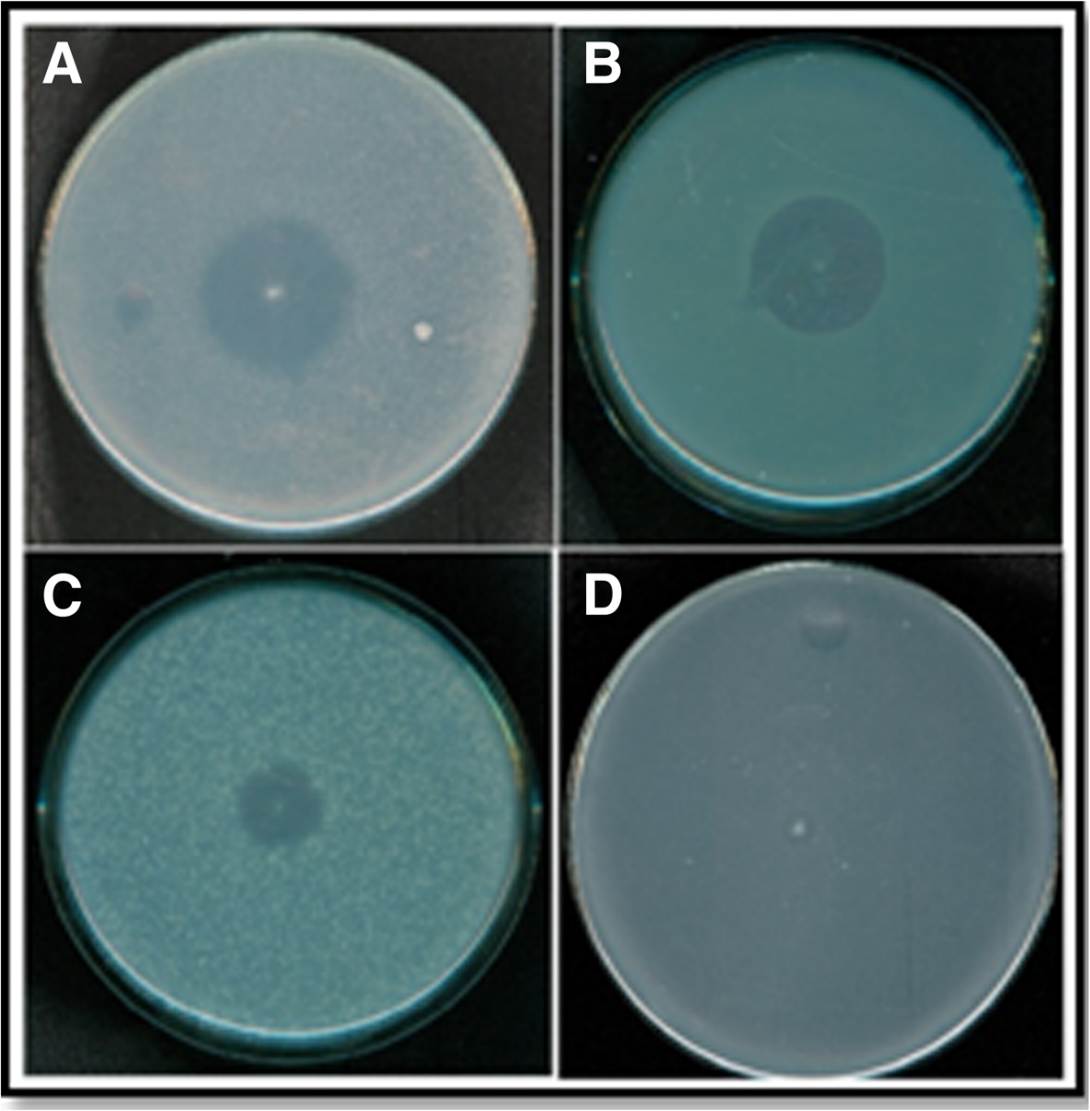
Antibacterial activity of BAC-IB17 against various microbes by stab and overlay method. **a** Methicillin resistant *Staphylococcus aureus* KIBGE-IB23; (**b**) Methicillin sensitive *Staphylococcus aureus* KIBGE-IB24; (**c**) *Bacillus stearothermophilus* KIBGE-IB29; (**d**: *Escherichia coli* ATCC 8739*.* All the strains were grown at 37 °C for 24 h on nutrient agar plates except *B. stearothermophilus* which was grown at 55 °C

Different bacterial strains (*n* = 40) were isolated and screened for antimicrobial activity against Gram’s positive and Gram’s negative microorganisms. In the current study, various strains of MRSA and MSSA were also isolated and identified. The 16S rDNA gene sequences of highly sensitive strains were deposited in GenBank and received following accession numbers: Methicillin resistant *Staphylococcus aureus* KIBGE-IB23 [KC465400] and Methicillin Sensitive *Staphylococcus aureus* KIBGE-IB24 [KC465401]. A phylogenetic tree for both the organisms was constructed and compared with the previously reported sequences available in NCBI GenBank database. 16S rDNA gene sequence showed 89 to 100% identity within the current isolates and the previously reported sequences retrieved from GenBank database. Figure 1 demonstrated the broad antibacterial potential of BAC-IB17 against different organisms using stab and overlay method.

### Purification and molecular weight estimation of BAC-IB17

**Fig. 2:**
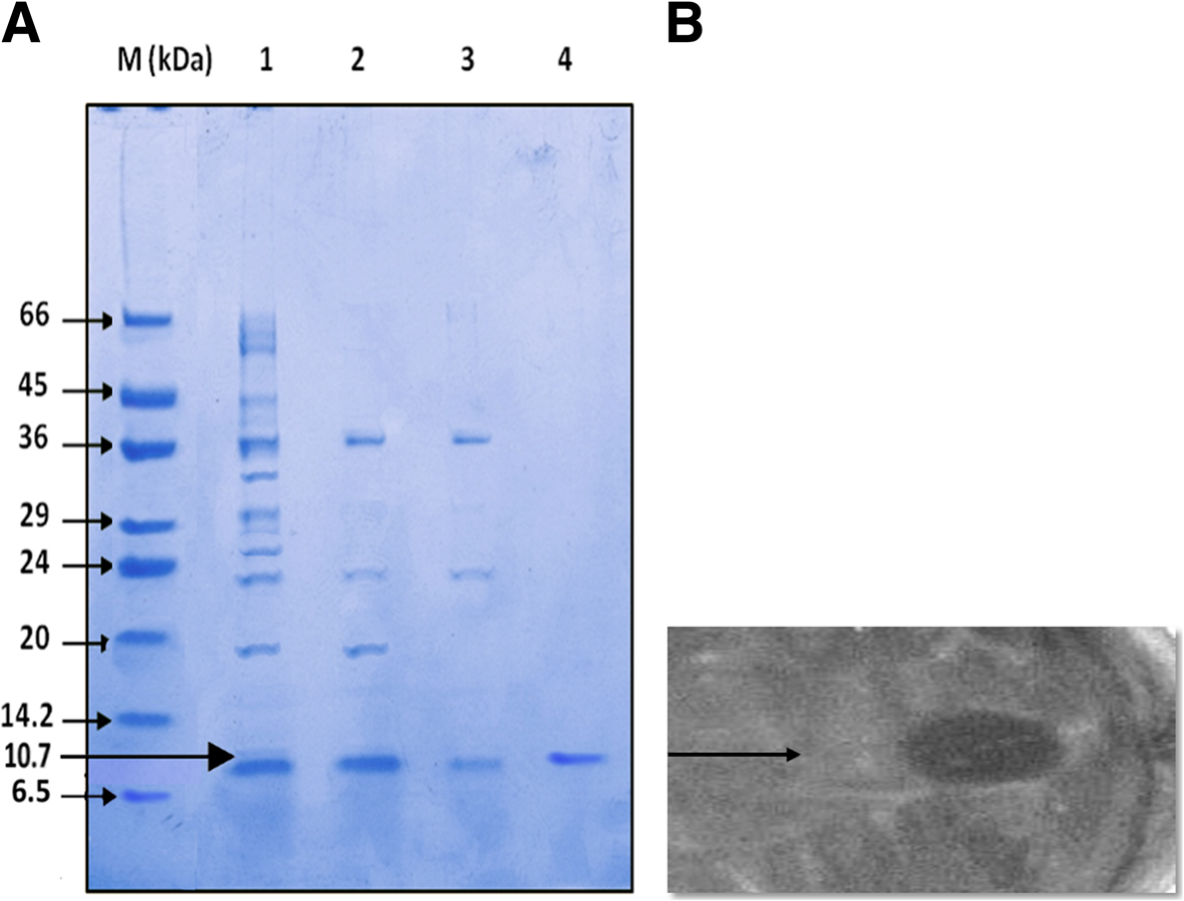
**a** SDS-PAGE Profile of BAC-IB17 produced by *Bacillus subtilis* KIBGE-IB17. Lane M: Low molecular weight markers (6500-66,000, Sigma-Aldrich, USA). Lane 1: BAC-IB17 precipitates after (NH_4_)_2_SO_4_ saturation. Lane 2: BAC-IB17 precipitates after passing through PD-10 desalting column. Lane 3: BAC-IB17 precipitates after passing through Centricon® ultrafilter device. Lane 4: Purified and concentrated BAC-IB 17 after passing through Sepahrose-CL6B column. **b** Gel Overlay Assay of Band of interest against MRSA

BAC-IB17 was partially purified using 40% ammonium sulphate saturation and desalted through PD-10 desalting columns with an increase in the arbitrary units, fold purification and specific activity from 2.5 to 5.72 times and 166.29 to 380.95 (AU mg^− 1^). After passing through Centricon centrifugal filter device (cutoff: 10.0 and 30.0 kDa) the antibacterial activity was detected in the retainate and then in filtrate, respectively. Fractions collected from CL-6B column were pooled and concentrated. Final specific activity and fold purification increased from 130 to 186 AU mg^− 1^ and 15.6 to 22.3 times, respectively with maximum arbitrary units of 1505.88 AU mg^− 1^. Tricine SDS-PAGE was also performed after each step of purification, which revealed that the estimated molecular weight of purified BAC-IB17 was approximately 10.7 kDa with the specific activity of band of interest in gel overlay assay (Fig. 2 and b). The arbitrary units and the minimal inhibitory concentration of the purified BAC-IB17 were 80 AU ml^− 1^ and 50 μg ml^− 1^, respectively.

### BAC-IB17 mode of action

**Fig. 3:**
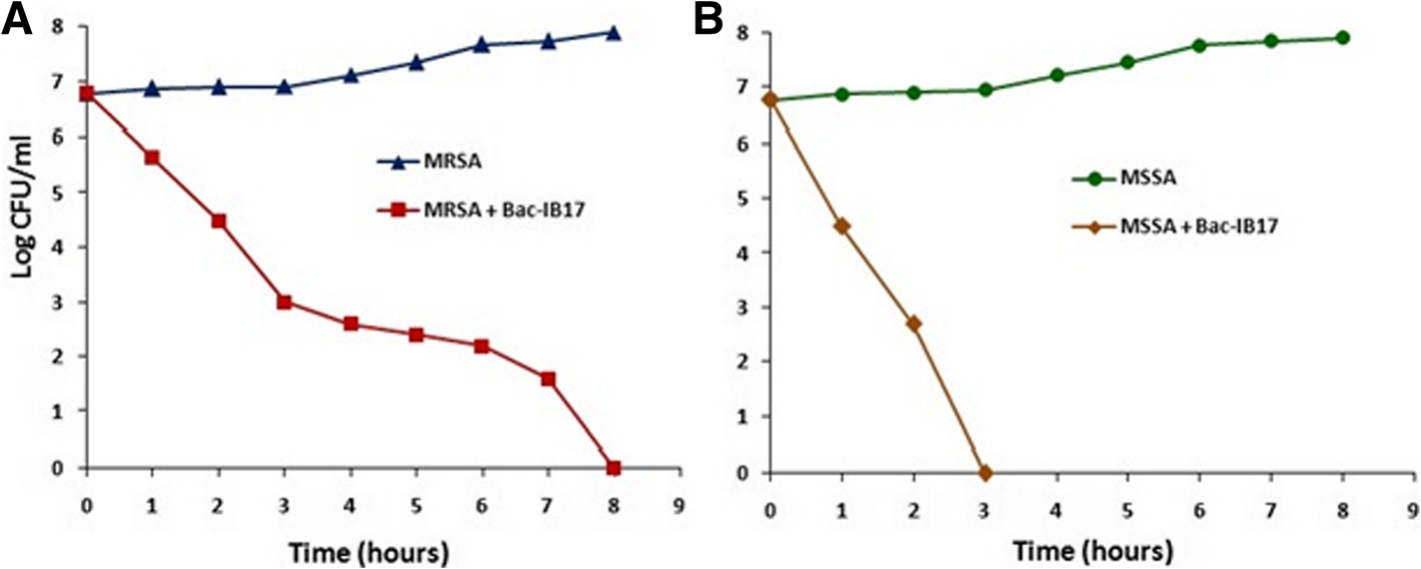
Bactericidal effect of BAC-IB17 on the growth and survival of MRSA and MSSA. **a** Methicillin resistant *Staphylococcus aureus*; (**b**) Methicillin sensitive *Staphylococcus aureus*

Killing kinetics revealed that purified BAC-IB17 exhibits a bactericidal mode of action against the tested indicator organisms in a lethal concentration of 80 AU ml^− 1^. For this purpose, the rate of survival of two indicator organisms including MRSA (KIBGE-IB23) and MSSA (KIBGE-IB24) after the treatment with BAC-IB17 was monitored using plate counting method. In case of MRSA, complete growth reduction was observed in 8.0 h whereas, for MSSA it was 3.0 h only. However, for further confirmation of bactericidal mode of action, the regrowth of both the organisms was monitored up to 24 h (Fig. 3).

### Influence of physical and chemical parameters on BAC-IB17

The influence of temperature, pH and various metal ions were also detected on the antibacterial potential of BAC-IB17 (Table 2). BAC-IB17 was found to be highly thermostable as it retained 100% of its antibacterial activity at 60 °C up to 3.0 h whereas; only 25% loss in activity was detected after 4.0 h of exposure at 80 °C. However, BAC-IB17 retained 50% of its activity at a broad range of pH. At acidic pH value (pH 5.0) the antibacterial activity was completely lost however, 60% of its antibacterial activity was detected at pH 6.0. Table 2 demonstrated that 100% relative activity was observed at physiological pH and bacteriocin was stable at alkaline pH values as compared to the acidic site. Moreover, the effect of pH on this bacteriocin was independent of the exposure time. It was also observed that upon long-term storage at − 20 °C, no loss in antibacterial activity of BAC-IB17 was detected even after 01 year of storage (data not shown). BAC-IB17 was also stable in the presence of various metal ions of alkaline-earth cation series. Metal Ions exhibited stabilizing effect on antibacterial potential of bacteriocin in low concentration whereas, Co^2+^ and Hg^2+^ completely inhibited the antibacterial activity.

**Table 2:** Antibacterial activity of BAC-IB17 against methicillin resistant *Staphylococcus aureus* after treatment with various physical and chemical parameters

Treatment	% Relative Antibacterial Activity				Treatment	Antibacterial Activity (AU ml^− 1^)			
**Control**	100				**Control**	80			
**a. Temperature (°C)**	**Exposure Time (hours)**								
	1.0	2.0	3.0	4.0	**d. Surfactants** ^**b**^				
40	100	100	100	100					
60	100	100	100	96	Tween 20 (10 mM)	160			
80	97	83	80	75	Tween 80 (10 mM)	160			
100	70	50	nil	nil	EDTA (100 mM)	160			
**b. pH**	**Exposure Time (minutes)**				SDS (01 mM)	160			
	15.0	30.0	60.0	120.0	Triton X-100 (0.25 mM)	nil			
5.0	nil	nd	nd	nd	**e. Organic Solvents** ^c^				
6.0	60	60	60	60					
7.0	100	100	100	100	DMSO (100 mM)	160			
8.0	80	80	80	80	Ethanol (05 mM)	160			
9.0	75	75	75	75	Methanol (100 mM)	80			
**c. Metal Ions** ^a^	**Concentration (mM)**				Formaldehyde (20 mM)	80			
	1.0	5.0	10.0		Chloroform (100 mM)	40			
Ba^+ 2^	100	100	100		Isopropanol (100 mM)	20			
Cu^+ 2^	100	nil	nd		**f. Enzymes** ^d^	37 °C		50 °C	
Mn^+ 2^	100	90	90			02 h	24 h	02 h	24 h
Cs^+ 2^	100	90	90		Protease (0.5 U)	80	40	80	40
Ca^+ 2^	100	100	100		Proteinase K (20.0 U)	80	40	80	40
Mg^+ 2^	95	95	90		Pepsin (15.0 U)	80	40	80	40
Ni^+ 2^	80	80	80		Lysozyme (150,000 U)	40	nil	40	nil
Zn^+ 2^	70	30	nil		Amylase (30.0 U)	40	nil	40	nil
Co^+ 2^	60	nil	nd		Ribonuclease A (90.0 U)	80	80	80	80
Hg^+ 2^	nil	nd	nd		Catalase (11,000 U)	80	80	80	80

*nd* Not determined

*nil* No antibacterial activity

^a^Metal ions used as chloride salts

^b^None of the surfactants alone (−ve control) in the above mentioned concentration inhibited the indicator strain except Triton X-100

^c^None of the organic solvents alone (−ve control) in the above mentioned concentration inhibited the indicator strain

^d^Final Concentration of each enzyme: 1.0 mg

**Fig. 4:**
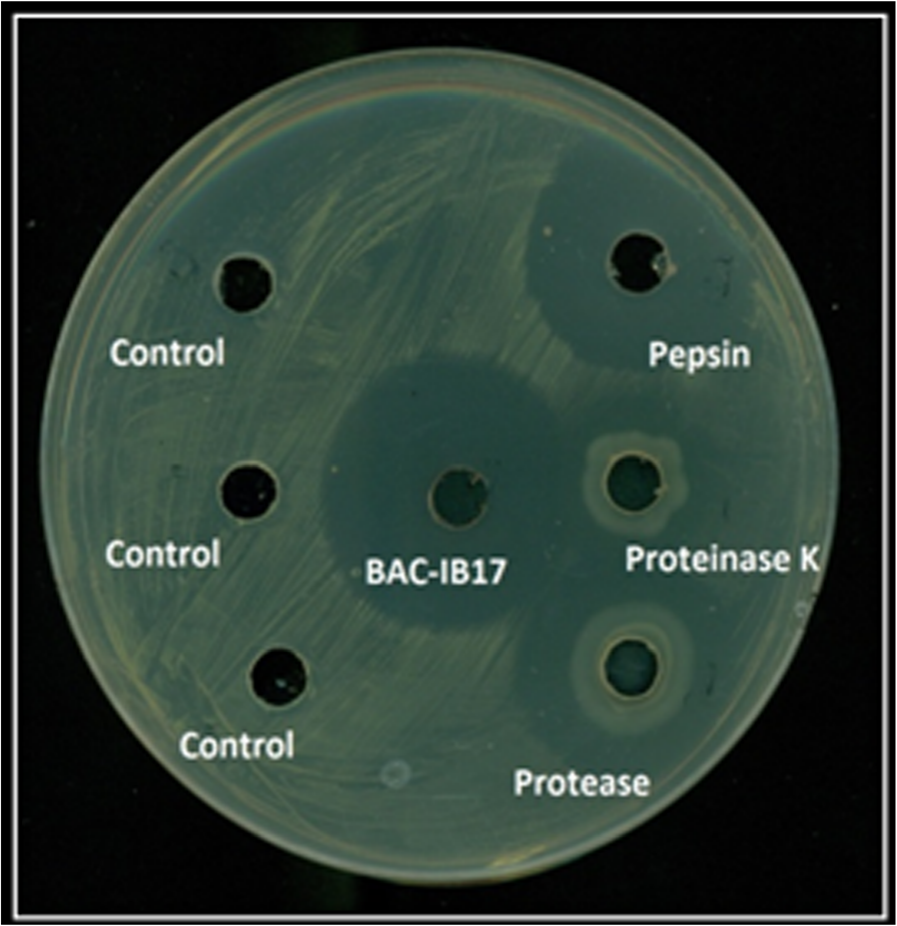
Effect of different proteolytic enzymes on the antibacterial activity of BAC-IB17. Control: all the respective enzymes alone were used as a negative control

Antibacterial activity of BAC-IB17 was increased in the presence of various surfactants. Two-fold increase in the activity was observed after treatment with Tween 20, Tween 80, EDTA and SDS. However, Triton X-100 completely inhibited the indicator strain itself even at low concentration (0.05 mM) and produced no effect on the antibacterial activity of BAC-IB17. Among various organic solvents tested, DMSO and ethanol exhibited an accelerating effect whereas, methanol and formaldehyde stabilized the inhibitory effect of this bacteriocin. Chloroform and isopropanol suppressed its antibacterial effect. Although bacteriocins are generally sensitive to proteolytic enzymes, resistance of some low molecular weight peptides to various proteases was not an unusual feature. Therefore, the effect of proteolytic enzymes on BAC-IB17 revealed that BAC-IB17 was completely resistant to various proteolytic enzymes up to 2.0 h but 50.0% loss in antibacterial activity was observed when the exposure time was extended up to 24 h (Fig. 4). While in case of lysozyme and amylase, complete loss in antibacterial activity was detected after 24 h. Ribonuclease A and catalase had no profound effect on BAC-IB17 (Table 2).

### MICs of BAC-IB17 and different antibacterial drugs

In order to determine the clinical effectiveness of BAC-IB17, its efficacy was compared with several known anti-MRSA and broad-spectrum drugs. It was observed that indigenously isolated MRSA (KIBGE-IB23) was resistant to some commercially available broad-spectrum antibiotics including ciprofloxacin, oxacillin and ertapenem however, sensitive to BAC-IB17 in the minimal inhibitory concentration of 50 μg ml^− 1^. Furthermore, MICs of other drugs were also determined against indigenously isolated MRSA (Table 3).

**Table 3:** Minimal Inhibitory Concentrations (MICs) of BAC-IB17 and commercially available antibiotics against Methicillin Resistant *Staphylococcus aureus* (MRSA)

Antibacterial Drugs	Minimal Inhibitory Concentrations (μg mL^−1^)
BAC-IB17	50
Cefepime	30
Linezolid	15
Ampicillin	10
Vancomycin	2
Ceftriaxone	2
Teicoplanin	1
Clindamycin	1
Ertapenem	R
Ciprofloxacin	R
Oxacillin	R

Key: *R* Resistant

### Amino acid analysis and N-terminal sequencing

Compositional amino acids analysis of BAC-IB17 represented a unique distribution profile based on only thirteen amino acids that were identified using standard amino acid solution (Sigma) with reference to retention time. Compositional analysis of amino acid showed that BAC-IB17 consist of both polar (Arginine, Aspartc acid, Glutamic acid, Threonine, Lysine and Serine) and non-polar (Alanine, Methionine, Leucine, Glycine, Tyrosine, Valine and Proline) amino acids. Furthermore, the N-terminal sequence identification of first 15 residues of pure BAC-IB17 was revealed that this bacteriocin constitutes a unique sequence: NH_2_-Asn-Lys-Pro-Glu-Ala-Leu-Val-Asp-Tyr-Thr-Gly-Val-X-Asn-Ser (NKPEALVDXTGVXNS). Whereas; X indicates an amino acid for which the identity could not convincingly be established [TrEMBL/UniProtKB/SwissProt database: C0HJE5].

## Discussion

In the past few years the emerging cases of multidrug resistance due to the overuse and misuse of antibiotics has enforced the use of new antimicrobials for combating infections. Threat posed to public health by various multidrug resistant (MDR) organisms can be resolved by discovering new antimicrobials with possibility to antimicrobial resistance. The current study suggests that BAC-IB17 can be used as an alternative therapeutic agent in the prevention and treatment of infections caused by MRSA.

Bacteriocin BAC-IB17 was obtained after several steps of purification. After purification, the understanding of biochemical nature and kinetic behavior of bacteriocin will be significantly assisted in its classification. For this different physicochemical parameters were considered for the characterization of BAC-IB17. Bacteriocins have wide range of molecular weight therefore; gradient system using tricine SDS-PAGE was selected for the determination of molecular weight of BAC-IB17 and the estimated molecular weight was 10.7 kDa. Several other bacteriocins have also been separated using tricine SDS-PAGE system including a broad spectrum bacteriocin known as paracin-1.7 (11.0 kDa) produced by *Lactobacillus paracasei* HD1.7, a new bacteriocins Bacthuricin F103 (11.0 kDa), entomocin 9 (12.4 kDa) from *B. thuringiensis* and Bac 14B (20 kDa) from *Bacillus subtilis* [19–22]. Killing kinetics was performed to determine the bactericidal and/or bacteriostatic effect against sensitive strains used. Results suggests that BAC-IB17 has a bactericidal mode of action which could be due to the increase in the permeability of the cytoplasmic membrane of the indicator strains that allows the release of hydrophilic molecules from the pore complexes resulting in cell death. [23, 24].

BAC-IB17 was found to be highly stable under different physicochemical conditions. Unambiguous reason for the thermal stability of BAC-IB17 is unknown however; there could be many factors responsible in providing stability to proteins at higher temperatures. High concentration of proline residues, salt bridges, hydrogen bonds and polar surface residues can reduce the risk of instability of any protein at elevated temperatures therefore similar factors might be involved in providing thermal stability to BAC-IB17. These factors not only stabilize the proteins but are also responsible for regulating its kinetics at higher temperatures [25]. The stability of a bacteriocin against various metal ions might be due to the induction of conformational changes in the protein structure, as some metal ions worked as cofactors and rendered proteins stability in soluble form [26]. However in case of Co^2+^ and Hg^2+^, the complete loss of antibacterial potential of BAC-IB17 might be due to the blockage of active sites. Furthermore, surfactants accelerated the effect of bacteriocin, normally surfactants unfold proteins by affecting three-dimensional confirmations of proteins and they can also increase the solubility of various proteins due to which the activity of the protein enhances [27]. On the contrary, different organic solvents exerted both the accelerating and the suppressing effect on BAC-IB17. Both these effects could be due to the changes in the conformational structure of this protein as a result of modifications in non-covalent interactions. Stability of BAC-IB17 in the presence of organic solvents could be supportive in a manner that most of the organic solvents are commonly used in preparation of stock solutions for various drugs specifically DMSO. In this perception, BAC-IB17 could be a plausible candidate for the treatment of skin infections caused by various strains of MRSA. Resistance of BAC-IB17 to various proteases was not an unusual feature as some low molecular weight peptides were also found to be resistant to proteolytic enzymes [28]. This effect may be due to the presence of cyclic peptides containing unusual amino acids at the active site of bacteriocin [29]. In case of lysozyme and amylase, BAC-IB17 completely lost its antibacterial activity suggesting that there could be some glycosidic moieties present in BAC-IB17 which may possibly responsible for its biological activity. Similar studies on sublancin (glycopeptides) also showed that glycosylation is essential for its antibacterial activity [30]. Another bacteriocin produced by *Lactobacillus curvatus* SE1 was reported containing some carbohydrate moiety essential for its activity as its activity decreases in the presence of amylolytic enzymes [31]. However, Ribonuclease A and catalase have no profound effect on BAC-IB17. When the MIC of BAC-IB17 was compared with the MICs of other anti-MRSA drugs, it was observed that the MICs of the commercially available drugs are low as compared to the MIC of BAC-IB17. Due to the continuous increased in the drug resistance, it is suggested that BAC-IB17 could be a better alternative antimicrobial agent to treat MRSA infections as it is naturally isolated from a microorganism.

Amino acid compositional analysis revealed presence of proline; a distinctive cyclic amino acid mainly present in thermostable proteins and responsible for the resistance of proteins against high temperatures. The comparison of the alignment of N-terminal amino acid sequence of BAC-IB17 with reference to other bacteriocins revealed that no homology exists with the already existing sequence of bacteriocins from various *Bacillus* species. Therefore, it is concluded that BAC-IB17 is a novel bacteriocin. This dissimilarity could be due to the presence of inconsistent amino acid sequences of different bacteriocins because one bacterium can produce multiple bacteriocins at a time.

## Conclusions

Keeping in view, the current data of the BAC-IB17 with reference to its biochemical properties and the sequence, it is concluded that the BAC-IB17 does not belong to the class I, II and III as proposed by Abriouel et al. [32]. As it is thermostable in nature and contain some glycosidic moiety responsible for its activity, may be it belongs to Class IV of the bacteriocin [33, 34]. Continuous research on diversified bacteriocins will increase their applications in the health and microbial control alternatives [35]. After reviewing potential properties of BAC-IB17, its plausible applications could be in various pharmaceutical and food industries as it can function under a variety of harsh environmental conditions.

## Abbreviations

AU: Arbitrary units
CFU: Colony forming units
DMSO: Dimethyl sulfoxide
MDR: Multidrug resistance
MRSA: Methicillin resistant *Staphylococcus aureus*
MSSA: Methicillin sensitive *Staphylococcus aureus*
SDS: Sodium dodecyl sulphate
UV: Ultra violet
